# On the Pressure Response in the Brain due to Short Duration Blunt Impacts

**DOI:** 10.1371/journal.pone.0114292

**Published:** 2014-12-05

**Authors:** Christopher W. Pearce, Philippe G. Young

**Affiliations:** College of Engineering, Mathematics and Physical Sciences, University of Exeter, Harrison Building, North Park Road, Exeter, EX4 4QF, United Kingdom; Tel Aviv University, Israel

## Abstract

When the head is subject to non-penetrating (*blunt*) impact, contusion-type injuries are commonly identified beneath the impact site (the *coup*) and, in some instances, at the opposite pole (the *contre-coup*). This pattern of injury has long eluded satisfactory explanation and blunt head injury mechanisms in general remain poorly understood. There are only a small number of studies in the open literature investigating the head's response to short duration impacts, which can occur in collisions with light projectiles. As such, the head impact literature to date has focussed almost exclusively on impact scenarios which lead to a quasi-static pressure response in the brain. In order to investigate the response of the head to a wide range of impact durations, parametric numerical studies were performed on a highly bio-fidelic finite element model of the human head created from *in vivo* magnetic resonance imaging (MRI) scan data with non-linear tissue material properties. We demonstrate that short duration head impacts can lead to potentially deleterious transients of positive and negative intra-cranial pressure over an order of magnitude larger than those observed in the quasi-static regime despite reduced impact force and energy. The onset of this phenomenon is shown to be effectively predicted by the ratio of impact duration to the period of oscillation of the first ovalling mode of the system. These findings point to dramatically different pressure distributions in the brain and hence different patterns of injury depending on projectile mass, and provide a potential explanation for dual *coup*/*contre-coup* injuries observed clinically.

## Introduction

Every year in the United States around 1.7 million people sustain traumatic brain injury (TBI), and of these 52,000 die. For the year 2000 alone, the direct medical cost and indirect cost through loss of productivity resulting from this high TBI rate was estimated at $60 billion [1]. TBI is commonly caused by impact to the head, which can be broadly classified as being either *blunt* or *penetrating* depending on whether or not the cranial vault is breached. As opposed to penetrating injuries, the mechanisms of blunt impact injury are not fully understood and cannot be well predicted [2].

The vast majority of blunt head impact studies, whether analytical, numerical or cadaveric, are limited to a narrow range of contact durations, typically from 3 to 10 ms [3]–[7]. In cases such as these it has previously been shown that the resulting intra-cranial pressures are predominantly caused by the rigid-body acceleration of the skull [8], [9]. The dilatational wave speed for grey and white matter is large (essentially that of water) so that a pressure pulse can typically traverse the diameter of the cranial cavity 10 times per millisecond [9] and, as such, the pressure response for impacts above 3 ms will be essentially hydrostatic (n.b. *quasi-static*); a linear pressure gradient will be induced in the brain, varying from a maximum positive pressure (*P_quasi_*) under the site of impact (*coup*) to a minimum negative pressure (*–P_quasi_*) at the *contre-coup*. These peak internal pressures can be predicted quite straightforwardly from the expression *P_quasi_ = r_c_·ρ·F_max_/m*, where *r_c_* is the distance measured from the centre of gravity of the brain to its exterior surface at the *coup* or *contre-coup*, *ρ* is the density of brain tissue, *F_max_* is the peak force transmitted by the impact, and *m* is the total mass of the head [8]; a quasi-static response is reported almost without exception in experimental and numerical head impact modelling literature [3]–[7], [10].

There is a surprising paucity of research on short duration head impacts: while a few studies exist which probe impacts beneath 3 ms in duration [8], [10]–[15], to the authors' knowledge the only published works which investigate impacts below 1 ms in duration are early experimental studies on fluid-filled shells by Kenner & Goldsmith [12] and Kabo & Goldsmith [13], numerical studies by Wahi & Merchant [14] and Young & Morfey [8], and analytical work by Young [15]. A probable reason for this is that previous studies have principally focussed on the head striking a fixed or heavy object, such as a windscreen or the ground, and it can be shown that these scenarios will invariably lead to comparatively long duration collisions [15]. However, insult by light projectiles will give rise to shorter contact times; an expression for the impact duration of an idealised two-phase model of the head (a homogeneous isotropic spherical shell filled with inviscid fluid) colliding with a spherical projectile has been proposed [15], in which it can straightforwardly be shown that impact duration is close to linearly proportional to the square root of m*, where the quantity m* is determined by the ratio of projectile mass to head mass and is given by the expression *m_head_·m_projectile_/(m_head_+m_projectile_)*. In the present study a highly bio-fidelic model, both in terms of geometry and material properties, is used to explore the dynamic pressure response of the brain as a consequence of a short duration impact. Here blunt injury is investigated and we are concerned primarily with the brain's pressure response, as intra-cranial pressure is regarded as a good indicator of injury severity and has been linked to the onset of cerebral contusions [11], [16], i.e. focal lesions characterised by tissue rupture and cortical bleeding [17]–[19].

## Materials and Methods

In order to explore the intra-cranial pressure response to a range of blunt head impacts, simplified and accurate finite element models were generated based on an MRI scan of the head and a number of impacts were simulated using the LS-DYNA explicit finite element code (LSTC Inc.). Impact conditions were varied, principally impactor mass, in order to produce a wide range of contact durations.

### Data Acquisition

The investigation was divided into two stages, employing models of medium and high bio-fidelity. Both models were developed from high resolution T1 weighted MRI scan data, obtained *in vivo* from an adult male volunteer using a Philips Gyroscan Intera 1.5 T whole-body imager operated by a radiologist at the Exeter MR Research Centre, UK. The volunteer provided written informed consent, and the study was approved by the College of Engineering, Mathematics and Physical Sciences (CEMPS) Ethics Committee, University of Exeter. The scan progressed axially from the top of the head to the base of the neck, and had resolution of 1.03516 mm×1.03516 mm in the coronal plane, with slice-to-slice separation of 1.04001 mm. The volunteer was 26 years of age and had no diagnosed conditions; his height and weight were measured prior to the scan and were recorded as 1.8 m and 81 kg respectively, both of which are within 1.5% of the American 50^th^ percentile male [20].

A commercial image-based meshing code, ScanIP (Simpleware Ltd.), was used to identify and segment biological structures from the image data into labelled regions of interest (ROIs). The segmentation was performed using a combination of image threshold operations which distinguish regions of the image data based on greyscale intensity, and morphological filters which dilate or erode these ROIs. Where necessary, such as to avoid small overlaps between structures, some ROIs were edited manually using an image ‘painting’ approach. The regions distinguished in this fashion could then be meshed simultaneously using a technique adapted from the marching cubes method [21], so creating a conforming finite element model of the head and its internal structures. In this way, the use of primarily automated and semi-automated image-based meshing techniques allowed the complex biological structures of the head to be modelled to a high degree of geometric accuracy that would arguably be otherwise unobtainable.

A convergence study was performed to determine the optimum finite element mesh density for use in these models. The meshing approach used generates elements based on the resolution of the image data; the head model geometry was meshed seven times at different resolutions yielding models with a range of mesh densities. The finite element solution to the head model's response to short duration impact was found to have reached convergence for an image with resolution of 2 mm in all three Cartesian directions. This resulted in a mixed tetrahedral and hexahedral mesh, where the hexahedral elements had an edge length of 2 mm, and were of cubic proportions which is desirable in critical areas. To ensure accurate contact modelling between the head and impactor, a region of local mesh refinement was defined with mesh density progressively increasing towards the contact site. At the contact site mesh density was increased by a factor of 48, yielding elements with 0.55 mm edge length.

### Preliminary Model: Skull and Cranial Contents

The initial model approximated the human head as comprising of two structures, the skull and cranial contents. The model was meshed using the parameters determined above, and the completed mesh was composed of just over 1.8M linear elements. This two-phase model was assigned frequently used material properties equal to those in Engin's pioneering study [22]: the skull bone was represented by a linear elastic material with modulus of 13.79 GPa, Poisson's ratio of 0.25, and density of 2140 kg/m^3^. The interior volume of the skull was filled with an ‘elastic fluid’ material with no shear resistance, representing the brain and intra-cranial fluids. This had a bulk modulus of 2.18 GPa, and density of 1002 kg/m^3^.

Impacts were simulated against this two-phase head model through the collinear collision of the initially stationary head with a spherical impactor of radius 40 mm. This took the form of a parametric study consisting of 12 impact simulations, each with varying impactor characteristics, such that a wide range of contact durations *T_P_* were produced. The impactor was linear elastic with a modulus of 0.8 GPa and Poisson's ratio of 0.25, while its initial velocity was varied incrementally between 0.2 and 7.0 m/s. In order to reduce variations in impact force, for each case the impactor's mass was calculated such that its kinetic energy remained constant at 0.16 J. Impacts were performed in the posterior to anterior direction and, to minimise rotational accelerations, were aligned such that the axis of impact passed through the head's centre of gravity. The resulting contact durations *T_P_* and peak impact forces *F_max_* were measured for each impact case. The duration that the two colliding bodies were in contact was retrieved using the LS-DYNA post-processing code T/HIS: examining the force reported at the centremost point of the contact area on the head model's surface allowed the times of initiation and cessation of the contact to be clearly distinguished. A wide range of contact durations were recorded, measuring from 0.17 ms to 3.29 ms.

### Bio-fidelic Model: Whole Head and Neck

**Figure 1:**
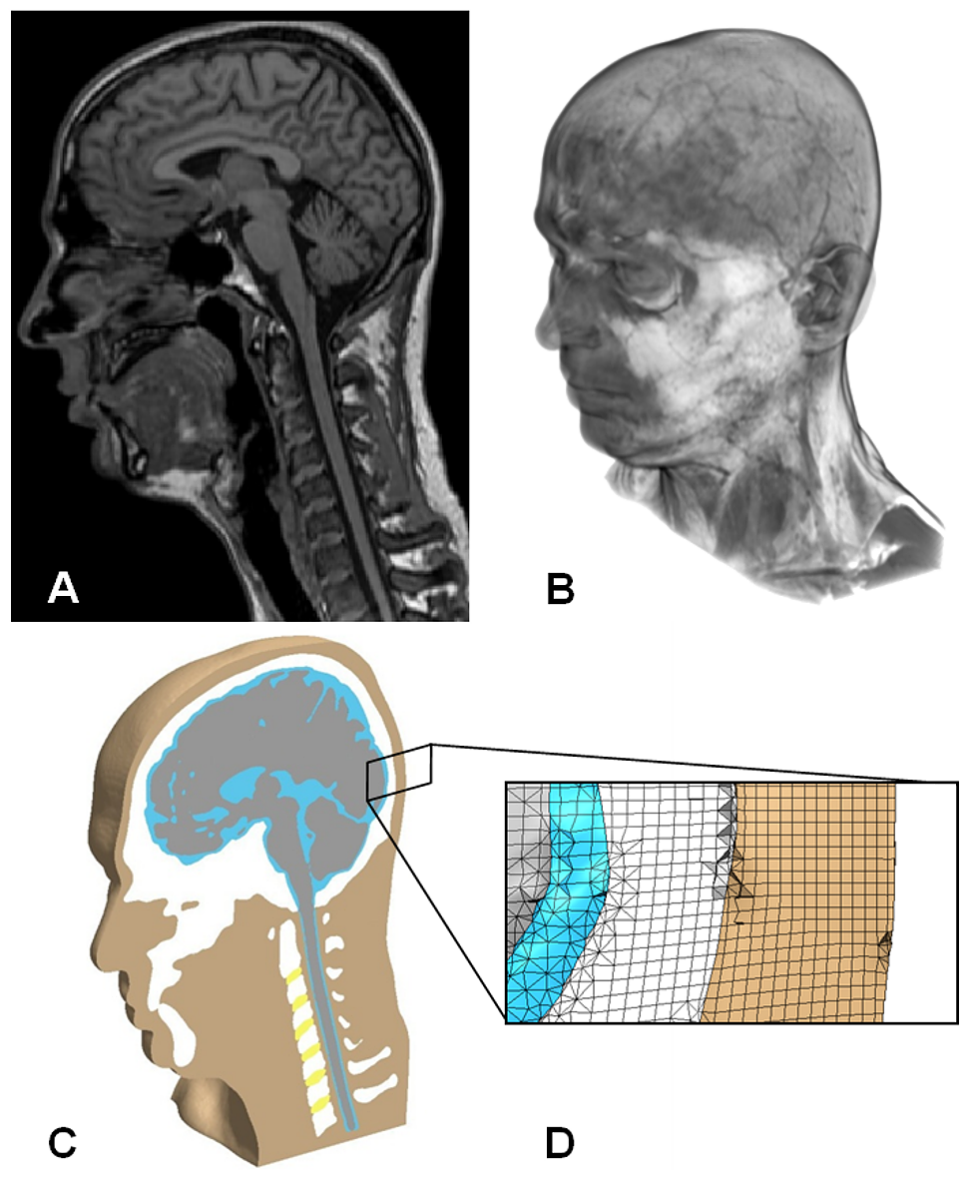
Image-based finite element model of the human head and neck. (**A**) Volume rendered image of MRI scan data used to construct this model. (**B**) Finite element head model subject to an example simulated golf ball impact at head posterior. (**C**) Isometric section view of model revealing soft and hard tissue structures. (**D**) Enlarged view of mesh at the occipital region of the head.

A sophisticated model of the whole head and neck was also developed which included the skull, mandible, cervical vertebrae, intervertebral discs, white matter, grey matter, spinal cord, cerebrospinal fluid (CSF), scalp, and surrounding flesh (Fig. 1). These structures were assigned properties as outlined in Table 1 following a thorough review of the literature. Many structures could be satisfactorily modelled by linear elastic materials, but those of particular importance to the impact response were assigned more complex material models: nervous tissue was represented by a viscoelastic model, the CSF was an elastic fluid, and the scalp beneath the impact site was assigned non-linear elastic properties based on *in vitro* impact tests [23]. The bio-fidelic finite element head model was meshed as above and contained 13.2 M elements.

**Table 1:** Constitutive models by biological structure.

Structure	Material formulation	Material constants	Additional information
Grey Matter, White Matter, Cerebellum, Brain Stem	viscoelastic	*G_∞_* = 170 kPa, *G_0_* = 530 kPa, *β* = 35 s^−1^, *B* = 2.19 GPa, *ρ* = 1080 kg/m^3^	*G(t)* = *G_∞_*+*(G_0_*−*G_∞_)e* ^−*βt*^
Skull, Vertebrae	elastic	*E* = 6.50 GPa, *ν* = 0.22, *ρ* = 1700 kg/m^3^	
Intervertebral Discs	elastic	*E* = 8.00E-03 GPa, *ν* = 0.35, *ρ* = 1140 kg/m^3^	
Cerebrospinal Fluid, Ventricles	elastic fluid	*B* = 2.19 GPa, *ρ* = 1006 kg/m^3^	zero shear resistance
Scalp, Flesh	elastic	*E* = 1.67E-02 GPa, *ν* = 0.42, *ρ* = 1200 kg/m^3^	
Scalp at impact site (non-linear)	user defined stress-strain relation [23]	*E* = variable, *ν* = 0, *ρ* = 1200 kg/m^3^	
Impactor	elastic	*E* = 0.10 GPa, *ν* = 0.49, *m_light_* = 10.0 g, *m_golf_* = 44.4 g, *m_heavy_* = 14.0 kg	

Table of material properties assigned to the segmented structures of the head model: grey matter, white matter, cerebellum and brain stem are assigned viscoelastic properties using the standard Flügge model [24]. The cerebrospinal fluid is represented by an inviscid fluid similar to water. The scalp is modelled as a non-linear solid, with a complete stress-strain curve as obtained from published impact tests [23]. The skull, mandible, cervical vertebrae and intervertebral discs are assigned linear elastic material properties.

The bio-fidelic model was validated against cadaveric experimental data by recreating impact trial 37 from Nahum et al's widely used benchmark paper [4]. In Nahum et al's experiment, stationary unembalmed cadavers were subjected to head impacts by rigid masses of constant velocity, and the pressure-time histories were monitored at various locations in the head. In accord with Nahum et al's methodology, here a simulated impact was delivered to the model's frontal bone at 45° to the Frankfurt anatomical plane. To ensure accurate reproduction of the experimental loading, the force-time history of the impact recorded in Nahum et al's experiment was applied directly to the finite element model. The simulation showed good agreement with the experimental intra-cranial pressures documented in Nahum et al's work, i.e. pressures at the frontal cortex and posterior fossa.

Following its validation, the bio-fidelic finite element model was used to explore the intra-cranial pressures resulting from three blunt head impact case studies. The model was subjected to three collisions by a spherical impactor with a radius of 21.39 mm, which is the size of a standard golf ball [25], and elastic modulus of 0.1 GPa, which has been shown to perform well as a homogeneous approximation to the stiffness of a golf ball's core [26]. As before, impacts were performed in the posterior to anterior direction and were aligned with the head's centre of gravity. Initially the impactor was assigned a density such that its mass was 44.4 g, which is approximately the mean mass of commercial golf balls [26], and an initial velocity of 76.0 m/s, which is just below the maximum allowable velocity a golf ball must not exceed under test conditions [27]. In this case the impactor was deliberately made similar to a golf ball in order to demonstrate a head injury scenario that may give rise to a short duration, blunt head impact. This resulted in the collision having an impact energy E_K_* of 127.4 J; here impact energy is defined as E_K_* = 0.5 m*·Δv^2^, where *Δv* is the mutual approach velocity of the two objects.

To explore a range of contact durations two further case studies were simulated. In the first (‘Heavy Impactor’), the mass of the object was increased to 14.0 kg, but its initial velocity reduced to 7.3 m/s such that impact energy remained constant at 127.4 J. In the final case study (‘Light Fragment’), the mass of the impactor was reduced to 10.0 g, while its velocity remained equal to that of the golf ball at 76.0 m/s, so resulting in an impact energy of 28.8 J.

## Results

### Preliminary Model: Parametric Study

The results of the preliminary investigation utilising the two-phase finite element head model are presented in Table 2. Throughout the parametric study, contact durations *T_P_* and peak impact forces *F_max_* were collated and pressures throughout the brain were examined. Maximum positive and negative pressure was found invariably to occur in the *coup* and *contre-coup* regions of the brain, and values of peak positive and negative intra-cranial pressure arising in these locations were recorded for each impact case. This data took the form of parameters *P_C positive_*, *P_C negative_*, *P_CC positive_*, and *P_CC negative_*, which were respectively the positive and negative peak pressures captured at the *coup*, and the positive and negative peak pressures at the *contre-coup*.

**Table 2:** Results of parametric study.

Case number	Contact duration	Peak impact force	Pressure at *coup*		Pressure at *contre-coup*	
	*T_P_* (ms)	*F_max_* (kN)	*P_C positive_* (MPa)	*P_C negative_* (MPa)	*P_CC positive_* (MPa)	*P_CC negative_* (MPa)
1	3.287	0.470	1.230e-2	−1.830e-3	2.771e-4	−1.197e-2
2	1.681	0.873	2.540e-2	−5.830e-3	1.390e-3	−2.151e-2
3	1.076	0.957	3.420e-2	−1.940e-2	7.555e-3	−2.435e-2
4	0.786	0.975	5.510e-2	−2.290e-2	7.048e-3	−3.087e-2
5	0.620	0.978	1.003e-1	−5.790e-2	3.174e-2	−3.720e-2
6	0.504	0.976	1.257e-1	−8.770e-2	5.690e-2	−4.560e-2
7	0.432	0.977	1.253e-1	−1.130e-1	5.800e-2	−6.892e-2
8	0.371	0.976	1.500e-1	−1.320e-1	6.360e-2	−8.739e-2
9	0.329	0.975	1.730e-1	−1.455e-1	7.698e-2	−9.756e-2
10	0.295	0.974	1.960e-1	−1.860e-1	8.583e-2	−1.170e-1
11	0.208	0.968	2.860e-1	−3.535e-1	1.014e-1	−1.609e-1
12	0.169	0.960	3.480e-1	−4.730e-1	1.036e-1	−1.643e-1

Impact conditions, and resulting peak positive and negative pressures recorded at both *coup* and *contre-coup* using the image-based, finite element model of the skull and cranial contents.

In agreement with the quasi-static theory, the magnitude of the intra-cranial pressures for comparatively long duration impacts (approximately 1 ms and above) was independent of the measured contact duration *T_P_* and, at any time *t*, solely a function of the instantaneous force applied *F(t)*. However, for shorter duration impacts (*T_P_*<1 ms), the maximum pressures in the brain increased significantly above those predicted by the quasi-static solution and large fluctuating pressure transients were observed in both the *coup* and *contre-coup* regions.

The numerical results were non-dimensionalised to reduce the influence of any variation in impact force, and allow qualitative changes in the pressure response to be revealed more clearly. The recorded peak intra-cranial pressures at the *coup* and *contre-coup* were normalised over the analytically predicted peak pressures *P_quasi_* and *–P_quasi_*, i.e. the pressure that would be expected at either the *coup* or *contre-coup* for a quasi-static response under the same peak force. In addition, contact durations *T_P_* were normalised over the period of oscillation of the first (n = 2) mode of vibration of the head model *T_Ω_*.

### Bio-fidelic Model: Individual Case Studies

**Figure 2:**
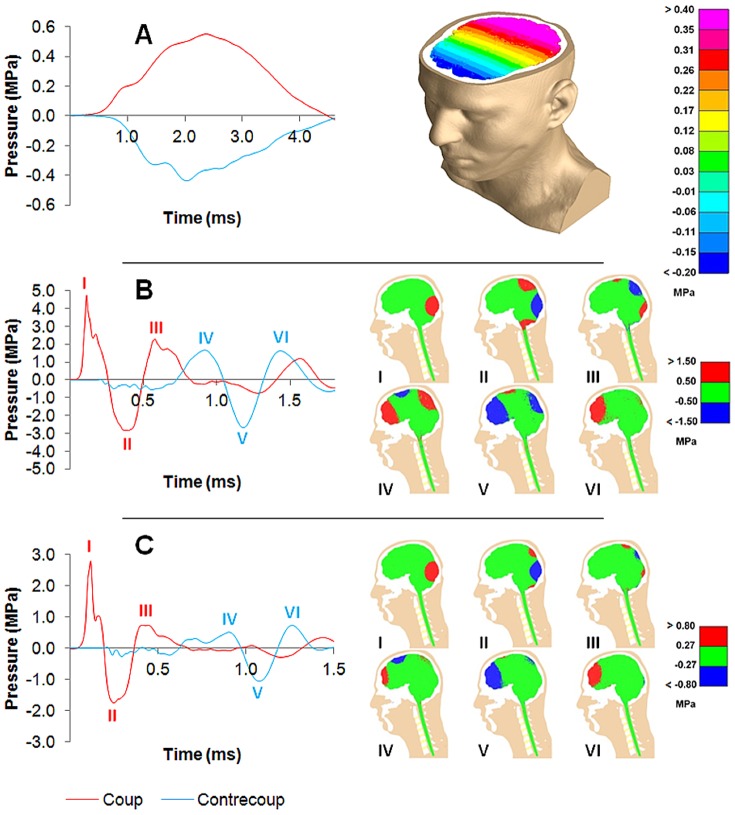
Intra-cranial pressure-time histories and distributions. Pressure-time histories at the *coup* (red lines) and *contre-coup* (blue lines) for impacts with different mass projectiles, and contour plots captured at different instants in each case. (**A**) Impact with heavy projectile (14.0 kg at 7.3 m/s; impact energy E_K_* = 127.4 J): a quasi-static pressure-time history is observed. (**B**) Impact with golf ball (44.4 g at 76 m/s; identical impact energy as heavy projectile E_K_* = 127.4 J): dynamic pressure-time history observed at *coup* and *contre-coup*. (**C**) Impact with light fragment (10.0 g also at 76 m/s; impact energy E_K_* = 28.8 J): again dynamic pressure-time history is observed.

Three case studies were carried out using the full bio-fidelic head model impacted with identical golf ball sized projectiles of different masses (‘Heavy Impactor’ 14.0 kg, ‘Golf Ball’ 44.4 g, and ‘Light Fragment’ 10.0 g) and the engendered pressures in the brain were computed. Fig. 2 presents the pressure-time histories captured at the *coup* and *contre-coup* regions of the brain during these impacts; landmark points on these plots are marked by Roman numerals I-VI, which relate to contour plots I-VI depicting intra-cranial pressure at these times.

The ‘Heavy Impactor’ collision had contact duration of 4.65 ms with a peak impact force of 18.9 kN. The intra-cranial pressures agreed with the quasi-static theory: *coup* and *contre-coup* pressures rose and fell in proportion to the impact force, and were approximately equal and opposite (Fig. 2a). Remarkably different pressure distributions throughout the brain were observed for the two lighter impactors compared to the heavy impactor. For the ‘Golf Ball’ impact contact duration reduced to 0.53 ms with a peak impact force of 22.3 kN, whereas in the ‘Light Fragment’ case contact duration reduced further to 0.33 ms with a peak force of 6.95 kN. Here dynamic pressure-time histories were observed at the *coup* and *contre-coup* (Fig. 2b, c) characterised by large fluctuating pressure transients similar to those recorded in the parametric study. Also, in spite of the fact that the impact energies for the ‘Heavy Impactor’ and the ‘Golf Ball’ cases were identical, the resultant peak positive pressure observed at the *coup* was nine times larger and the peak negative pressure over six times larger at the *contre-coup* for the ‘Golf Ball’ impact. In this way, impact by a golf ball represents a realistic head injury scenario in which this counter-intuitive dynamic *magnification* of intra-cranial pressures can occur.

**Figure 3:**
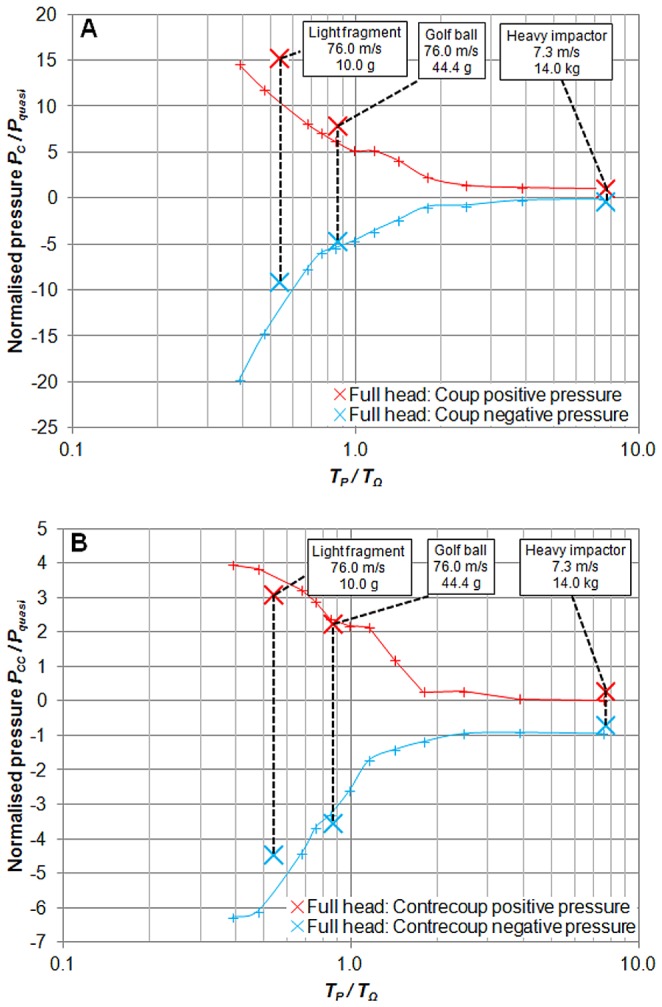
Normalised peak positive and negative intra-cranial pressures. (**A**) Non-dimensionalised pressures against non-dimensionalised impact durations at the *coup*, and (**B**) at the *contre-coup*. Solid lines represent results of the parametric study conducted using the simpler two-phase head model, and bold markers represent three case studies utilising the full bio-fidelic model.

As before, the recorded peak intra-cranial pressures were normalised over the analytically predicted peak pressures ±*P_quasi_*, so resulting in a measure of pressure magnification, i.e. the factor by which the pressure response of a particular short duration head impact is increased in comparison to a conventional quasi-static head impact with equal peak force. Impact durations *T_P_* were normalised over the period of oscillation of the first equivoluminal mode of vibration of the head *T_Ω_*. The normalised results for a range of impacts are presented in Fig. 3 in terms of non-dimensional pressure against the log of non-dimensional duration at both the *coup* and *contre-coup*. The results of the three case studies using the full bio-fidelic head model can be seen to exhibit non-dimensional pressure magnifications against impact duration which agree well with those of the simpler parametric study. The non-dimensional ratio *T_P_*/*T_Ω_* neatly collapses the system's response: above a value of approximately *T_P_*/*T_Ω_* = 2 the pressure distribution in the brain will be quasi-static and below this value increasingly large positive and negative pressure transients are observed. It is noteworthy that the most commonly adopted measure of head impact severity, the Head Injury Criterion (HIC) [28], correlates decidedly poorly with the observed pressure magnitudes in the brain; indeed the light fragment and golf ball impacts have low computed HIC values of 52 and 835 respectively, whilst the heavy impactor HIC score is 2650, yet in the case of the two lighter impactors the resulting peak positive pressures were observed to be between 5.3 to 8.8 times greater and negative pressures were 4.0 to 6.5 times greater.

Also of note is that the large dynamic pressure transients observed at the *coup* and later at the *contre-coup* (Fig. 2b, c) are not carried through the brain as a dilatational wave, as might be expected, but travel by way of antisymmetric Lamb waves propagating through the skull. Both a travelling positive pressure region followed immediately by a negative region can be observed radiating away from the point of impact, engendering rolling high positive and negative pressure fluctuations around the brain's circumference which converge at the *contre-coup* (see contour plots I-VI in Fig. 2b, c). The velocity of the zero-order antisymmetric Lamb wave mode (the *flexural* wave) was found to agree with the velocity which could be back-inferred from the transmission time between the arrival of the dynamic pressure transients at the *coup* and at the *contre-coup* taking into account the geodesic distance between these locations through the skull. Nodal velocities within the skull also revealed the tell-tale elliptical displacement patterns characteristic of antisymmetric Lamb wave motion.

## Discussion

In summary, qualitatively and quantitatively different pressure responses are observed in the brain depending on the impact duration. The onset of a dynamic response can be accurately predicted by the ratio of the impact duration to the period of oscillation of the first ovalling mode. For short duration impacts, the negative pressure pulses of high magnitude observed in this study suggest that transient cavitation [2], [3], [29]–[32] may be a possible mechanism for cerebral tissue damage not just at the *contre-coup*, but also at the *coup* site. Dynamic pressure transients are shown to be carried around the skull, from *coup* to *contre-coup*, by flexural waves, which may explain the peculiar distribution of trauma observed in dual *coup*/*contre-coup* injuries [2], [33]–[35]. The dynamic pressure magnification behaviour was observed in the highly bio-fidelic model despite the inclusion of the pressure relieving mechanisms of the foramen magnum and ventricular system, and other damage mitigating structures such as the cerebrospinal fluid and the thick layer of scalp tissue. The results indicate the importance of considering short duration impact, and strongly suggest the human head is susceptible to dangerous intra-cranial pressure transients during impacts which satisfy the condition *T_P_*/*T_Ω_* <2. This can occur in realistic head injury scenarios, as demonstrated by several case studies involving impact with light projectiles and such non-penetrating impacts can occur in a number of situations (impacts in sports, ballistic injuries with rubber bullets, assaults with bottles, stones, etc.). Head Injury Criterion (HIC) scores were shown to be completely at odds with the observed intra-cranial pressure response. Impacts with significantly lower HIC scores resulted in higher observed pressures, suggesting the HIC to be an unsuitable metric when considering short duration impacts (i.e. when a dynamic rather than quasi-static response is induced). These findings have important implications for injury prevention as well as in forensic investigation of blunt head injuries, as different patterns of trauma could be expected depending on the impact scenario.

## Supporting Information

File S1 **Detailed tabulated information pertaining to the simulated head impacts.** Impact characteristics and intra-cranial pressures recorded during impact simulations using the simpler two-phase head model and the bio-fidelic head model.
(DOC)
