# Supplementary material for: On the Pressure Response in the Brain due to Short Duration Blunt Impacts
Source: PLoS One. 2014 Dec 5;9(12):e114292. doi: 10.1371/journal.pone.0114292 (PMC4257587; doi:10.1371/journal.pone.0114292)
Supplement: File S1 — Detailed tabulated information pertaining to the simulated head impacts. Impact characteristics and intra-cranial pressures recorded during impact simulations using the simpler two-phase head model and the bio-fidelic head model. (DOC) [file pone.0114292.s001.doc]

# On the Pressure Response in the Brain due to Short Duration Blunt Impacts

# Authors

Christopher W. Pearce, Philippe G. Young

# File S1: Detailed tabulated information pertaining to the simulated head impacts

## Preliminary Model of Skull and Cranial Contents: Parametric Study

Tabulated information below contains data pertaining to the parametric study involving the preliminary head model composed of the skull and cranial contents.

**Table S1.** Impact variables (impactor initial velocity *v_sol_* and mass *m_sol_*) used in the parametric study.

| **Case number** | ***v_sol_* (m/s)** | ***m_sol_* (kg)** |
| --- | --- | --- |
| 1 | 0.2 | 8.0000 |
| 2 | 0.6 | 0.8889 |
| 3 | 1.0 | 0.3200 |
| 4 | 1.4 | 0.1633 |
| 5 | 1.8 | 0.0988 |
| 6 | 2.2 | 0.0661 |
| 7 | 2.6 | 0.0473 |
| 8 | 3.0 | 0.0356 |
| 9 | 3.4 | 0.0277 |
| 10 | 3.8 | 0.0222 |
| 11 | 5.4 | 0.0110 |
| 12 | 7.0 | 0.0065 |

**Table S2.** Resulting contact durations (*T_P_*) and peak impact forces (*F_max_*) in the parametric study.

| **Case number** | **Contact duration** | **Peak impact force** |
| --- | --- | --- |
|  | ***T_P_* (ms)** | ***F_max_* (kN)** |
| 1 | 3.287 | 0.470 |
| 2 | 1.681 | 0.873 |
| 3 | 1.076 | 0.957 |
| 4 | 0.786 | 0.975 |
| 5 | 0.620 | 0.978 |
| 6 | 0.504 | 0.976 |
| 7 | 0.432 | 0.977 |
| 8 | 0.371 | 0.976 |
| 9 | 0.329 | 0.975 |
| 10 | 0.295 | 0.974 |
| 11 | 0.208 | 0.968 |
| 12 | 0.169 | 0.960 |

**Table S3.** Analytically predicted quasi-static peak pressures at the *coup* (*P_C quasi_*) and *contre-coup* (*P_CC quasi_*) for the parametric study.

| **Case number** | **Analytical** | |
| --- | --- | --- |
|  | ***P_C quasi_* (MPa)** | ***P_CC quasi_* (MPa)** |
| 1 | 1.154e-2 | -1.284e-2 |
| 2 | 2.144e-2 | -2.386e-2 |
| 3 | 2.387e-2 | -2.616e-2 |
| 4 | 2.432e-2 | -2.665e-2 |
| 5 | 2.438e-2 | -2.672e-2 |
| 6 | 2.434e-2 | -2.668e-2 |
| 7 | 2.435e-2 | -2.668e-2 |
| 8 | 2.435e-2 | -2.668e-2 |
| 9 | 2.431e-2 | -2.664e-2 |
| 10 | 2.427e-2 | -2.660e-2 |
| 11 | 2.415e-2 | -2.646e-2 |
| 12 | 2.394e-2 | -2.624e-2 |

**Table S4.** The peak positive and negative pressure values recorded at both the *coup* and *contre-coup* during the parametric study.

| **Case number** | **Pressure at *coup*** | | **Pressure at *contre-coup*** | |
| --- | --- | --- | --- | --- |
|  | ***P_C positive_* (MPa)** | ***P_C negative_* (MPa)** | ***P_CC positive_* (MPa)** | ***P_CC negative_* (MPa)** |
| 1 | 1.230e-2 | -1.830e-3 | 2.771e-4 | -1.197e-2 |
| 2 | 2.540e-2 | -5.830e-3 | 1.390e-3 | -2.151e-2 |
| 3 | 3.420e-2 | -1.940e-2 | 7.555e-3 | -2.435e-2 |
| 4 | 5.510e-2 | -2.290e-2 | 7.048e-3 | -3.087e-2 |
| 5 | 1.003e-1 | -5.790e-2 | 3.174e-2 | -3.720e-2 |
| 6 | 1.257e-1 | -8.770e-2 | 5.690e-2 | -4.560e-2 |
| 7 | 1.253e-1 | -1.130e-1 | 5.800e-2 | -6.892e-2 |
| 8 | 1.500e-1 | -1.320e-1 | 6.360e-2 | -8.739e-2 |
| 9 | 1.730e-1 | -1.455e-1 | 7.698e-2 | -9.756e-2 |
| 10 | 1.960e-1 | -1.860e-1 | 8.583e-2 | -1.170e-1 |
| 11 | 2.860e-1 | -3.535e-1 | 1.014e-1 | -1.609e-1 |
| 12 | 3.480e-1 | -4.730e-1 | 1.036e-1 | -1.643e-1 |

**Table S5.** Non-dimensional peak positive and negative pressures, and non-dimensional impact durations, of the parametric study.

| **Case number** | **Normalised** | | | | |
| --- | --- | --- | --- | --- | --- |
|  | ***P_C positive_  / P_C quasi_*** | ***P_C negative_  / P_C quasi_*** | ***P_CC positive_  / P_CC quasi_*** | ***P_CC negative_  / P_CC quasi_*** | ***T_p_  / T_Ω_*** |
| 1 | 1.066 | -0.159 | 0.022 | -0.932 | 7.516 |
| 2 | 1.185 | -0.272 | 0.058 | -0.901 | 3.845 |
| 3 | 1.433 | -0.813 | 0.289 | -0.931 | 2.461 |
| 4 | 2.266 | -0.942 | 0.264 | -1.158 | 1.797 |
| 5 | 4.114 | -2.375 | 1.188 | -1.392 | 1.417 |
| 6 | 5.164 | -3.603 | 2.133 | -1.709 | 1.153 |
| 7 | 5.146 | -4.641 | 2.174 | -2.583 | 0.988 |
| 8 | 6.161 | -5.422 | 2.384 | -3.276 | 0.849 |
| 9 | 7.115 | -5.984 | 2.889 | -3.662 | 0.753 |
| 10 | 8.075 | -7.663 | 3.227 | -4.399 | 0.674 |
| 11 | 11.845 | -14.640 | 3.832 | -6.081 | 0.475 |
| 12 | 14.535 | -19.756 | 3.949 | -6.260 | 0.387 |

## Bio-fidelic Model: Individual Case Studies

Tabulated information below contains data pertaining to the individual case studies performed using the full bio-fidelic head model.

**Table S6.** Impact variables (impactor initial velocity *v_sol_* and mass *m_sol_*) used in the bio-fidelic model impact case studies.

| **Case study** | ***v_sol_* (m/s)** | ***m_sol_* (kg)** |
| --- | --- | --- |
| Impact by a golf ball | 76.0 | 0.0444 |
| Impact by a heavy spherical mass | 7.32 | 14.0000 |
| Impact by a light spherical mass | 76.0 | 0.0100 |

**Table S7.** Resulting contact durations (*T_P_*) and peak impact forces (*F_max_*) in the bio-fidelic model impact case studies.

| **Case study** | **Contact duration** | **Peak impact force** |
| --- | --- | --- |
|  | ***T_P_* (ms)** | ***F_max_* (kN)** |
| Impact by a golf ball | 0.526 | 22.285 |
| Impact by a heavy spherical mass | 4.653 | 18.851 |
| Impact by a light spherical mass | 0.326 | 6.948 |

**Table S8.** Analytically predicted quasi-static peak pressures at the *coup* (*P_C quasi_*) and *contre-coup* (*P_CC quasi_*) for the bio-fidelic model impact case studies.

| **Case study** | **Analytical** | |
| --- | --- | --- |
|  | ***P_C quasi_* (MPa)** | ***P_CC quasi_* (MPa)** |
| Impact by a golf ball | 6.152e-1 | -7.513e-1 |
| Impact by a heavy spherical mass | 4.938e-1 | -6.472e-1 |
| Impact by a light spherical mass | 1.918e-1 | -2.334e-1 |

**Table S9.** The peak positive and negative pressure values recorded at both the *coup* and *contre-coup* during the bio-fidelic model impact case studies.

| **Case study** | **Pressure at *coup*** | | **Pressure at *contre-coup*** | |
| --- | --- | --- | --- | --- |
|  | ***P_C positive_* (MPa)** | ***P_C negative_* (MPa)** | ***P_CC positive_* (MPa)** | ***P_CC negative_* (MPa)** |
| Impact by a golf ball | 4.907 | -2.865 | 1.690 | -2.651 |
| Impact by a heavy spherical mass | 5.550e-1 | -1.270e-1 | 1.870e-1 | -4.410e-1 |
| Impact by a light spherical mass | 2.938 | -1.744 | 7.230e-1 | -1.040 |

**Table S10.** Non-dimensional peak positive and negative pressures, and non-dimensional impact durations, of the bio-fidelic model impact case studies.

| **Case study** | **Normalised** | | | | |
| --- | --- | --- | --- | --- | --- |
|  | ***P_C positive_  / P_C quasi_*** | ***P_C negative_  / P_C quasi_*** | ***P_CC positive_  / P_CC quasi_*** | ***P_CC negative_  / P_CC quasi_*** | ***T_p_  / T_Ω_*** |
| Impact by a golf ball | 7.976 | -4.657 | 2.249 | -3.528 | 0.860 |
| Impact by a heavy spherical mass | 1.124 | -0.257 | 0.289 | -0.681 | 7.610 |
| Impact by a light spherical mass | 15.316 | -9.092 | 3.097 | -4.455 | 0.533 |
